# Barriers to Initiation of Pediatric HIV Treatment in Uganda: A Mixed-Method Study

**DOI:** 10.1155/2012/817506

**Published:** 2012-02-06

**Authors:** T. Sonia Boender, Kim C. E. Sigaloff, Joshua Kayiwa, Victor Musiime, Job C. J. Calis, Raph L. Hamers, Lillian Katumba Nakatudde, Elizabeth Khauda, Andrew Mukuye, James Ditai, Sibyl P. Geelen, Peter Mugyenyi, Tobias F. Rinke de Wit, Cissy Kityo

**Affiliations:** ^1^PharmAccess Foundation, P.O. Box 22700, 1100 DE, Amsterdam, The Netherlands; ^2^Department of Global Health, Academic Medical Center of the University of Amsterdam, Amsterdam Institute for Global Health and Development, P.O. Box 22700, 1100 DE, Amsterdam, The Netherlands; ^3^Joint Clinical Research Centre, Plot No. 101 Lubowa Hill, P.O. Box 10005, Kampala, Uganda; ^4^Global Child Health Group, Emma Children's Hospital, Academic Medical Center of the University of Amsterdam, P.O. Box 22660, 1100 DD Amsterdam, The Netherlands; ^5^Joint Clinical Research Centre, Pallisa Road, Mbale, P.O. Box 10005, Kampala, Uganda; ^6^Joint Clinical Research Centre, Kamwenge Road, Fort Portal, P.O. Box 10005, Kampala, Uganda; ^7^Department of Paediatric Immunology and Infectious Diseases, Wilhelmina Children's Hospital, University Medical Centre Utrecht, P.O. Box 85090, 3508 AB Utrecht, The Netherlands

## Abstract

Although the advantages of early infant HIV diagnosis and treatment initiation are well established, children often present late to HIV programs in resource-limited settings. We aimed to assess factors related to the timing of treatment initiation among HIV-infected children attending three clinical sites in Uganda. Clinical and demographic determinants associated with early disease (WHO clinical stages 1-2) or late disease (stages 3-4) stage at presentation were assessed using multilevel logistic regression. Additionally, semistructured interviews with caregivers and health workers were conducted to qualitatively explore determinants of late disease stage at presentation. Of 306 children initiating first-line regimens, 72% presented late. Risk factors for late presentation were age below 2 years old (OR 2.83, *P* = 0.014), living without parents (OR 3.93, *P* = 0.002), unemployment of the caregiver (OR 4.26, *P* = 0.001), lack of perinatal HIV prophylaxis (OR 5.66, *P* = 0.028), and high transportation costs to the clinic (OR 2.51, *P* = 0.072). Forty-nine interviews were conducted, confirming the identified risk factors and additionally pointing to inconsistent referral from perinatal care, caregivers' unawareness of HIV symptoms, fear, and stigma as important barriers. The problem of late disease at presentation requires a multifactorial approach, addressing both health system and individual-level factors.

## 1. Introduction

Despite the effectiveness of antiretroviral prophylaxis for the prevention of mother-to-child transmission (PMTCT) of HIV, approximately 370,000 children were newly infected with HIV in 2009. An estimated 2.5 million children are currently infected with HIV worldwide, of whom 2.3 million reside in sub-Sahara Africa [1]. HIV infected infants have much higher rates of disease progression and mortality than adults or older children, even with a relatively high percentage of CD4 T lymphocytes [2, 3]. Without treatment, over 50% of HIV-infected children are estimated to die before the age of two [4].

Despite the increased mortality in young infants, children in resource-limited settings generally initiate ART at an older age and with advanced disease [4, 5]. In 2008, the CHER trial in South Africa demonstrated a 76% mortality reduction among infants in whom antiretroviral treatment (ART) was initiated before 12 weeks of age, regardless of HIV symptoms or immunodeficiency, compared with those deferring therapy [6]. Based on these important findings, the World Health Organization (WHO) guidelines currently recommend that all HIV-infected children under the age of two should initiate treatment [7]. The WHO estimates that only 32% of HIV-infected children in East Africa requiring ART are currently treated [8].

 Although government policies and efforts by international donors seek to make antiretrovirals (ARVs) freely available to children through national ART programs, other factors are holding back further scale-up of pediatric ART in Africa. A wide array of such factors or barriers has been put forward in the literature including health system and personal level barriers [9, 10]. The development of new strategies to overcome these barriers is essential to reduce child morbidity and mortality, thereby contributing to the achievement of the Millennium Development Goal 4 [11]. However, limited structured research has been performed and there is little setting-specific insight into health care barriers.

The Joint Clinical Research Centre (JCRC) is a main provider of HIV care and treatment in Uganda. It was founded in 1990 as a strategic partnership with the Ministry of Health and Makerere University Medical School. The national JCRC network—consisting of more than 50 clinical sites—has over 20 years of experience with ART, from conducting clinical trials to nationwide roll-out programs since 2003. This study describes the characteristics of children initiating HIV care at three JCRC clinical sites based in Kampala, Fort Portal, and Mbale. Drawing on both quantitative and qualitative data sources, we aimed to identify the most important factors influencing the timing of pediatric ART initiation.

## 2. Methods

### 2.1. Population, Setting, and Study Design

We used an observational study design with a mixed methodology. This allowed us to triangulate findings from both participants and methods and generate a deeper understanding of the barriers to initiation of pediatric HIV care. The present study was performed as part of the Monitoring Antiretroviral Resistance in Children (MARCH) observational cohort, monitoring HIV-infected children (below 12 years old) initiating ART at three JCRC sites. The clinical sites in Kampala, Fort Portal, and Mbale are Regional Centers of Excellence and provide ART for both adult and pediatric patients. The Kampala site, based in the national capital, houses JCRC headquarters and mainly serves an urban population. The sites in Fort Portal and Mbale are located in district capitals, serving the Rwenzori region in Western Uganda and the entire Eastern region of Uganda, respectively [12]. People attending these two clinical sites come from both urban and rural areas (Table 1).

 The sample size was calculated based on the MARCH study objective to monitor HIV drug resistance. This cross-sectional, observational substudy aims to identify the most important factors influencing the timing of pediatric ART initiation at the three sites. Potential participants were informed of the study and screened for eligibility by the study staff at each clinic. All children that initiated ART were included; previous use of ARVs for the purpose of therapy (i.e., ART or mono/duo therapy) was an exclusion criterion. Previous use of ARVs for PMTCT was allowed. The ethical committees of JCRC and the Academic Medical Center of the University of Amsterdam approved the study protocol. The parent(s)/guardian(s) of all eligible children provided written informed consent. Children above the age of eight who were aware of their HIV status provided written informed assent. Routine sociodemographic, clinical, and laboratory data were collected using electronic case report forms, which were aggregated in a web-based data system. Whenever possible, the health status and medication use of the mother were also captured.

### 2.2. Quantitative Methods

Group comparisons for categorical data were performed using the chi-square test and for continuous data using Student's *t*-test. Nutritional status was assessed by means of the WHO Child Growth Standards: WHO Anthro version 3.2.2 (age 0–5) and WHO Reference 2007 for height and weight (age 5–19) [13, 14]. Severe immunodeficiency was classified according to the WHO guidelines: CD4 cell percentage <25% or CD4 cell count <1500 cps/mm^3^ below 12 months old; CD4 cell percentage <20% or CD4 cell count <750 cps/mm^3^ between 12 and 35 months old; CD4 cell percentage <15% or CD4 cell count <350 cps/mm^3^ above 35 months old [15]. WHO clinical staging was used to define early disease stage or late disease stage at presentation: children in stage 1 (asymptomatic) or 2 (mild symptoms) were considered to have early disease stage, and children in stage 3 (advanced symptoms) or 4 (severe symptoms) were considered to have late disease stage [16].

Multivariate logistic regression analysis with random intercepts was used to examine risk factors for late disease stage at presentation, while accounting for clustering of observations within sites. Results are expressed as odds ratios (ORs) with 95% confidence intervals (CIs) and *P* values, with two-sided *P* values < 0.05 considered statistically significant. A sensitivity analysis was performed, excluding children with unknown source of HIV infection. All analyses were performed with Stata version 10 (StataCorp LP, TX, USA).

### 2.3. Qualitative Methods

Qualitative semistructured interviews with both JCRC health workers and caregivers of children attending JCRC clinical sites were conducted to explore participants' views of key findings from the quantitative results, such as the late (disease stage) presentation of children for ART. Interviews consisted of open-ended questions to explore perceived barriers to ART initiation. Topics covered in the interview were the referral system, quality of care, HIV testing and treatment protocols, characteristics of the caregiver, transport to the clinic, and pharmacy and laboratory facilities. Pilot interviews were held with three local physicians; questions were adapted if necessary to ensure that they were appropriate for all participants. A separate questionnaire aimed at health workers, testing ART guideline knowledge, was developed in collaboration with a pediatric infectious disease specialist.

Interviews were conducted by the first author (TSB) in July 2011 at the three clinical sites. All caregivers of children below 12 years of age were identified by the doctor or counselor during a regular follow-up visit. Data were collected until the saturation point [17] was reached; we are therefore confident that the findings presented are internally valid. All health workers—pediatricians, clinicians, nurses, counselors, and adherence officers—involved in pediatric HIV care at JCRC were interviewed at all 3 clinics, to maximize health worker representation and internal validity.

All interviews took place in private settings where other people could not hear the respondents' answers. For interviews with caregivers, a local trained counselor assisted with translation and/or interpretation of questions. Interviews were recorded and transcribed in English. Using the framework approach for qualitative analysis [17], key issues and themes emerging from the data were identified, and responses were compared and contrasted among the different groups of study participants. Findings from the qualitative study were interpreted using Andersen's Behavioral Model of Health Services Use [18–20].

## 3. Results

### 3.1. Quantitative Results

#### 3.1.1. Participant Characteristics

Between January 2010 and May 2011, 310 children initiating first-line ART were enrolled in the MARCH study (92 from Kampala, 113 from Fort Portal, and 105 from Mbale). After excluding a protocol violation (*n* = 1) and children with missing data on eligibility criteria (*n* = 3), 306 participants were included in the analysis. The median age was 4.8 years and 50% (*n* = 152) were boys (Table 2). The reported source of HIV infection was mother-to-child transmission (MTCT) in 284 (93%) participants. In 22 (7%) children, the source of infection was unknown. HIV-status was known for 208 (68%) of their mothers, of whom 195 (94%) were HIV infected, 1 (0.5%) was uninfected, and 12 (6%) were unaware of their HIV status. Among the HIV infected mothers, 45% were on ART, 49% were not on ART, and for the remainder ART usage was unknown.

At first presentation, 72% of participants were in WHO clinical stage 3 or 4 (40% in Kampala, 82% in Fort Portal, and 90% in Mbale). Severe immunodeficiency according to a decreased CD4 cell count-for-age was present in 31% of children and in 51% when based on CD4 cell percentage-for-age. Severe immunodeficiency was more prevalent in Kampala compared to Fort Portal and Mbale (Table 2). There was a poor correlation between clinical staging and immunological status: of children in clinical stage 3 or 4, 47% also had severe immunodeficiency according to CD4 cell percentage-for-age.

In children below 5 years of age, the prevalence of weight-for-age *z*-score <−2 standard deviation (SD) was 43%; height-for-age *z*-score <−2 SD was found in 62% (Table 3). Of children in clinical stage 3 or 4, 70% had HIV-related malnutrition. The nutritional status of children did not differ significantly between the clinical sites.

#### 3.1.2. Risk Factors for Late Disease Stage at Presentation: Quantitative Results

Compared to children with early disease stage at presentation, children with late disease stage at presentation were more likely to be younger, to have an unemployed caregiver, and to have higher transportation costs. They were less likely to be living with both parents or to have a history of uptake of PMTCT services (Table 4). Late disease stage at presentation was not associated with sex, the caregiver's health status or education, transportation time, time between HIV-positive diagnosis and ART initiation, or waiting time at the clinic. The sensitivity analysis, excluding 22 children in whom MTCT was not confirmed, yielded similar associations (data not shown).

### 3.2. Qualitative Results

#### 3.2.1. Participant Characteristics

Interviews were conducted with 19 health workers and 30 caregivers. Twenty-one caregivers were HIV infected, six were uninfected, and three reported they were unaware of their status. Among the HIV-infected caregivers, sixteen caregivers were in care at JCRC and the remaining caregivers were in care at another clinic. Five health workers were specialized pediatricians; others were clinicians (*n* = 2), nurses (*n* = 6), counselors (*n* = 4), or adherence officers (*n* = 2) with pediatric training. The caregivers included those of children participating in the MARCH study and those of other clinic attendants aged 0–12 years old. All but four adults accompanying a child to the clinic were the primary caregivers. The caregivers were mostly self-employed; six were unemployed.

#### 3.2.2. Health System Factors: Resources and Organization

Kampala is the only site with a separate pediatric outpatient clinic. All sites have access to local laboratory facilities (including HIV-DNA PCR testing for infants <18 months of age), first- and second-line ARVs, and ready-to-use therapeutic food products (i.e., *Plumpy'nut*). According to the physician respondents, the clinic's capital and labor resources are sufficient to take care of all children attending the clinic. Occasional stock-outs of specific drugs or fixed-dose combinations were reported, in which case drugs are borrowed from other clinics or doctors prescribe different formulations to reconstruct the same regimen. When pharmacy stocks are low, ARV prescriptions are given for one month at a time rather than the regular three months.

All doctors were aware that ART should be initiated in infants below the age of two years, irrespective of CD4 count or clinical condition. The general consensus among health workers was that enough qualified personnel are available at the clinic, although the workload is high. There was often insufficient time for thorough counseling, which can result in longer waiting times for patients and caregivers attending the clinic. Health workers evaluated the pediatric HIV care delivered at JCRC as better in comparison to other clinics. However, health workers also noted that it is necessary to spread information about the clinic in the community.

*When we sit here and wait for people to come, we can wait for a long time. We have to go out there and tell about the available services so they can choose to come*. Female counselor, Mbale.

Health workers reported that many children are referred to JCRC after visiting private clinics, local hospitals, and sometimes traditional healers or herbalists for recurrent infections. There are no antenatal care (ANC) services at JCRC, and therefore only HIV-infected pregnant women who are already attending the JCRC adult clinic are immediately linked with pediatric care. Referral from external ANC clinics is limited. In Fort Portal, the regional general hospital offering ANC is adjacent to the JCRC clinic, which facilitates referral of HIV-infected pregnant women.

Family-centered care is not routinely offered at JCRC, but health workers encourage parents to bring their other children and family members. Disclosure issues play a role as health workers construct a family tree of the HIV status of the family members and ask to bring in any children with unknown status. When children and caregivers come for HIV testing, they receive a ticket with a number to match their test results. By using this method, people are assured of anonymous testing, thereby reducing fear of disclosure. Health workers' recommendations for improving access to care are listed in the Box 1.

#### 3.2.3. Population Factors: Living Situation and Transport

Both health workers and caregivers reported that children without parents are living under poorer conditions and present later in care. When the mother is receiving HIV care, the child is more likely to present early. The effect of living in an institution (e.g., orphanage) can go both ways. Some institutions bring their children early because they recognize the importance of HIV testing and can provide transport; others have fewer resources and do not prioritize HIV testing. Health workers pointed out that caregivers' financial constraints and lack of employment are important barriers to access health care. More highly educated caregivers seem to visit earlier, but taking time off work can be an obstacle. Some caregivers fear to disclose to their employers, and thus have a hard time justifying their absence to attend clinic visits. The unemployed have more time, but lack the money to visit the clinical sites.

*When the caregiver is employed, they have the advantage of money for transport, but they can be too busy at work to come. For the unemployed it is difficult to pay for transport. *Female counselor, Fort Portal.

Transportation costs are prohibitively high when considered in comparison to the need for food. Furthermore, having to travel long distances to reach the clinic makes it difficult for people to leave and return home within one day, especially when no or limited public transport is available. This problem was more frequently reported in Fort Portal and Mbale compared to Kampala.

*To some people transportation costs matter. For the ones living in the villages; they do not have the income, but they do have time*. HIV-positive mother of a five-year-old boy, Fort Portal.*My daughter lived in the villages and it was too far for her. That is why I am taking care of her daughter now. The mother is dead now. Just being a housewife, raising money for transport is hard. *HIV-negative grandmother of a ten-year-old girl, Mbale.

#### 3.2.4. Population Factors: Knowledge, Stigma, and Fear

According to health workers, many women are delivered by traditional birth attendants and are not tested for HIV during pregnancy. This increases the likelihood that a child's infection remains unnoticed; the child may only be tested after becoming clinically ill or after the loss of one or both parents. Caregivers have often visited other clinics for the child's frequent infections before enrolling at the JCRC clinic. Health workers described that health-seeking behavior among caregivers can be delayed due to lack of knowledge or denial of HIV symptoms. Caregivers also reported that HIV is something people do not think about or do not want to think about. They are often not ready to disclose their or their child's HIV status to others. Health workers recommended involving men more actively in ANC, in order to improve the uptake of PMTCT measures and enrolment of HIV-exposed children in pediatric HIV care.

*Men need more involvement, include men to PMTCT, now only very few come. They are the biggest decision makers in the home. This would strengthen adherence too*. Female pediatric counselor, Fort Portal.

Health workers explained that sensitization campaigns by means of radio and advertisements were helpful to spread information about HIV prevention and care and decrease stigma. At the same time, they acknowledge that stigma experienced by caregivers remains an important barrier to care.

*HIV is not a taboo anymore. There used to be a lot of stigma, in the '90 and early '00. There's been a lot of sensitization for HIV. You see more and more people test and seek care*. Male doctor, Mbale.*HIV is not a taboo anymore, but there's stigma. People do not want to associate with HIV. They fear to come to the clinic because someone might see them, which affects adherence. Stigma also delays the start of ART*. Female counselor, Fort Portal.

Fear was a major factor reported by caregivers as a barrier to visiting ART clinics. People fear to be seen at the clinic and fear that other people get to learn about their HIV status. Fear of disclosure appeared to be more common in the smaller towns compared to the city, as evidenced by the responses from both health workers and caregivers.

*I never got married and feared to tell my mother. She is harsh and will tell everybody to stigmatize me. Nobody knows about my and my daughter's HIV status. They discriminate you, even at work. *HIV-positive mother of a three-year-old girl, Mbale.

## 4. Discussion

This mixed-method study examined factors influencing the timing of ART initiation among children attending HIV clinics in Uganda. Even though ART is now free and widely available in Uganda, 72% of the children in this study presented with advanced HIV disease at their initial visit. The main risk factors for this late disease stage at presentation identified in our study—from both quantitative and qualitative data—included lack of HIV-specific perinatal care, living without parents, financial constraints of the caregiver, caregivers' unawareness of HIV symptoms, stigma, and fear. Our study adds insight into the challenges of identifying HIV-infected infants and children sooner and recruiting them into care. In the setting of the JCRC network of HIV treatment sites in Uganda, linkage to the ANC systems and psychosocial support are recognized as priorities to improve pediatric access. Even though JCRC sites are at the high-end with respect to resources, infrastructure, staff, and available diagnostics, late disease stage at presentation was a frequent and important problem among children initiating ART. The barriers identified in our study are therefore likely of national relevance and applicable to other HIV clinics in Uganda.

### 4.1. Health System Factors

The linkage between ANC and pediatric ART clinics was found to be inconsistent. This lack of coordination across services is similar to previous studies investigating barriers to timely pediatric ART initiation in resource-limited settings [22, 23]. Failure to diagnose HIV in pregnancy, to provide PMTCT services, and to followup the HIV-exposed infant represent missed chances for prevention of HIV transmission. As previous research has also shown, integrating antenatal services, PMTCT, early infant diagnosis, and pediatric HIV care greatly improves outcomes for HIV-infected infants in resource-limited settings [24–28].

As ANC is not performed at JCRC, this challenge could be addressed by closer collaboration between JCRC Centers of Excellence and outside ANC providers. HIV-infected women should be routinely referred to have their infants tested after delivery and actively followedup to ensure they receive the results. Health workers at JCRC have suggested collaborating with traditional birth attendants to reach women who do not visit regular ANC service centers. Additionally, improving male involvement in ANC was proposed as men could be decision-makers in seeking care for the child. Studies have shown that male attendance in ANC is a cost-effective strategy to increase PMTCT uptake and is associated with reduced MTCT and infant mortality [29–31].

We examined the time between HIV test and ART initiation and found that it was increased in older children. It is possible that the first HIV-positive test was performed outside of JCRC, with a subsequent referral delay for ART initiation. Secondly, children might have been tested at JCRC in early disease stage and started ART later when immunological or WHO stage criteria were met. This is in line with the high numbers of children in care at JCRC in whom ART is not yet initiated (Table 1). Regression analysis showed that lag time between the first HIV diagnosis and ART initiation was not a significant risk factor for late disease stage at presentation.

Although the clinics in Fort Portal and Mbale have similar resources as the Kampala clinic, the latter was found to have a higher percentage of early disease stage presenters. The clinic urban setting likely contributes to improved accessibility, and stigma was reported less frequently in the qualitative study compared to the other sites. Health workers at all JCRC clinics were well trained and consistently adhered to current pediatric HIV guidelines. The clinics could give more attention to community outreach and active case finding in order to increase parents' awareness and to identify HIV-infected children before the onset of symptoms. Community outreach could be targeted specifically at the most vulnerable children, such as those in orphanages. Alternatively, outreach could be performed by screening infants at immunization clinics [32].

Rates of underweight and stunted children were alarmingly high, which concurs with previous reports among HIV-infected children in Uganda [33, 34]. Malnutrition was a common clinical stage 3 or 4 defining symptom, and therefore timely referral to HIV care is perhaps the most critical nutritional intervention. JCRC routinely provides therapeutic foods. Additionally, nutritional education for caregivers and sufficient supply of micronutrients are important to decrease rates of underweight and stunting [35].

### 4.2. Population Factors

When examining individual-level barriers to care, the child's living situation was found to be an important determinant. This corresponds with earlier studies in which orphans were more likely to initiate ART at an older age with lower baseline CD4 levels and more advanced WHO staging [36, 37]. In addition, unemployment of the caregiver and high travel costs were risk factors for late disease stage at presentation in quantitative analysis. The qualitative interviews in our study confirmed these socioeconomic factors as important barriers, especially in the smaller towns. Prior studies from Uganda and other resource-limited settings have reported similar findings, suggesting that interventions such as community outreach, transportation refunds, home-based ART distribution, or outreach clinics in orphanages might be useful in overcoming these issues [38–42].

Other personal factors observed in the interviews were caregivers' unawareness of HIV symptoms, stigma, and fear, confirming that these personal beliefs discourage people from seeking ART [10, 22]. In addition to lack of knowledge of HIV symptoms, unawareness of free HIV services also impedes timely presentation [43]. Media campaigns designed to inform people about ANC, HIV testing, and free ART for children could be improved at relatively low cost [44, 45].

One of this study's strengths was the mixedmethod approach by which quantitative data could be contextualized and confirmed by qualitative study. Methodological triangulation increased the credibility of the findings and enhanced comprehensiveness of the study, creating a deeper understanding of the barriers to initiation of pediatric HIV care [46–49]. A limitation of the study is the cross-sectional design, making it difficult to establish causal relations in the quantitative analysis. In the qualitative study, there is a risk of socially desirable answers during the interviews.

Finally, different types of selection bias may have affected our study. First, late disease stage at presentation could have been overestimated as many children were referred to JCRC after visiting other clinics; the disease stage at presentation in the referring clinics was not part of our study. Second, our study did not take into account the HIV-infected children that died before reaching the clinic. As the median age was over 4 years old in our cohort, this population consisted of mostly medium and slow progressors. Younger children appeared to be a risk factor for late disease stage at presentation but this finding is subject to survival bias and should be interpreted accordingly. Additionally, other risk factors identified might not apply to children with fast disease progression. The specific barriers experienced by these children and their caregivers should be evaluated in longitudinal studies of HIV-exposed children.

In conclusion, although first-line ART has become widely available for HIV-infected children in Uganda, this alone does not ensure timely access. The problem of late disease stage at presentation requires a multifactorial approach, prioritizing community and orphanage outreach programs, and linkage of ANC systems to ART providers. Knowledge of these factors and their potential solutions is important in order to help health workers and ART program planners to create interventions to reach HIV-infected infants as early as possible and avoid preventable child mortality.

##  Authors' Contributors

C. Kityo, P. Mugyenyi and V. Musiime established the MARCH cohort and supervised data collection. K. C. E. Sigaloff and J. Kayiwa contributed to implementation. T. F. R. Wit, C. Kityo, K. C. E. Sigaloff, S. P. Geelen, J. Calis and T. S. Boender conceived the substudy. T. S. Boender analyzed the data and wrote the first draft of the manuscript with assistance from R. L. Hamers and K. C. E. Sigaloff. C. Kityo, V. Musiime, J. Calis, T. F. R. Wit and S. P. Geelen critically reviewed the paper. All authors contributed to subsequent drafts and reviewed and approved the final manuscript.

##  Conflict of Interests

There is no conflict of interests to declare.

## Figures and Tables

**Box 1:**
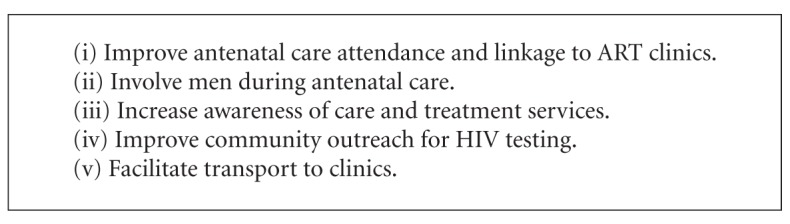
Health workers' recommendations to improve timely access to ART for children.

**Table 1:** Characteristics of the Joint Clinical Research Centre (JCRC) sites.

	Kampala	Fort Portal	Mbale
Location	National capital	District capital	District capital
Population^a^	1,659,600	47,100	91,800
Catchment area	Urban	Urban and rural	Urban and rural
HIV prevalence^b^	5–9.9%	5–9.9%	5–9.9%
Number of adults in care at JCRC (% of total)^c^	15306 (85.7)	5880 (87.3)	3027 (85.7)
Number of children in care at JCRC (% total)^c^	2553 (14.3)	858 (12.7)	506 (14.3)
Number of adults receiving ART at JCRC (% of adults in care)^c^	6096 (39.8)	2575 (43.8)	2505 (82.8)
Number of children receiving ART at JCRC (% of children in care)^c^	888 (34.8)	340 (39.6)	349 (69.0)

^a^Source: Uganda Bureau of Statistics (UBOS) 2011.

^b^Source: UNAIDS Epidemiological Factsheet Uganda 2009.

^c^Source: Monitoring and Evaluation records at JCRC, 2011.

**Table 2:** Clinical and demographic characteristics of the “Monitoring of Antiretroviral Therapy in Children” cohort participants.

		Overall	Study site			*P* value
		*n* = 306 (100)	Kampala *n* = 91 (29.7)	Fort Portal *n* = 112 (36.6)	Mbale *n* = 103 (33.7)	
Sex	Male	152 (49.8)	43 (47.3)	55 (49.1)	54 (52.9)	0.719

Age, median years (IQR)		4.8 (2.2–8.6)	4.0 (1.5–8.5)	4.2 (1.9–8.5)	5.8 (3.0–8.7)	0.127

Age groups	<2 years old	76 (24.8)	32 (35.2)	29 (25.9)	15 (14.6)	0.005
	2–5 years old	84 (27.5)	18 (19.8)	37 (33.0)	29 (28.2)	
	5–12 years old	146 (47.7)	41 (45.1)	46 (41.1)	59 (57.3)	

Age at (first) confirmed HIV+ test	Median (IQR)	3.7 (1.6–6.8)	3.0 (1.3–7.2)	3.3 (1.1–6.8)	4.5 (2.7–6.8)	0.264

WHO clinical stage	Stages 3 and 4	221 (72.2)	36 (39.6)	92 (82.1)	93 (90.3)	<0.001

HIV-TB coinfection	Pulmonary tuberculosis	31 (10.1)	18 (19.8)	6 (5.4)	7 (6.8)	0.001

Severe immunodeficiency^a^	CD4 count-for-age	67 (31.0)	36 (40.0)	9 (21.4)	22 (26.2)	0.047
	CD4 %-for-age	102 (51.3)	57 (63.3)	18 (42.9)	27 (40.3)	0.008

Viral load, median log_10_ cps/mL (IQR)^c^		5.0 (4.4–5.5)	5.2 (4.7–5.6)	5.1 (4.2–5.5)	4.7 (4.1–5.3)	0.001

Main reason for ART initiation^d^	HIV diagnosis <24 months	30 (9.8)	20 (22.0)	9 (8.0)	1 (1.0)	<0.001
	Immunological status	102 (33.3)	55 (61.4)	25 (22.3)	22 (21.4)	
	WHO clinical stage	174 (56.9)	16 (17.6)	78 (69.6)	80 (77.7)	

Time between HIV test and ART initiation, median days (IQR)	<2 years old	43 (19–85)	40 (18–66)	48 (23–103)	43 (26–86)	0.299
	2–5 years old	97 (26–400)	117 (34–309)	197 (29–546)	64 (15–180)	0.302
	5–12 years old	258 (29–802)	242 (28–634)	296 (35–757)	216 (21–848)	0.474

PMTCT exposed	Yes	14 (4.6)	11 (12.1)	3 (2.7)	—	<0.001

Drugs for PMTCT	Single dose NVP	9 (2.9)	6 (6.6)	3 (2.7)	—	0.025
	NVP	4 (1.3)	4 (4.4)	—	—	0.008
	AZT	2 (0.7)	2 (2.2)	—	—	0.093
	Unknown	1 (0.3)	1 (1.1)	—	—	0.306

Breastfeeding	Yes	24 (7.9)	10 (11.1)	12 (10.7)	2 (1.9)	0.023

“Is there enough food in the household?”	Yes	300 (98.0)	87 (95.6)	110 (98.2)	103 (100.0)	0.087

Data are presented as *n *(%) unless otherwise indicated.

^a^Severe immunodeficiency, defined as CD4 percentage < 25% or CD4 count < 1500 cps/mm^3^ below 12 months old, CD4 percentage < 20% or CD4 count < 750 cps/mm^3^ between 12 and 35 months old, and CD4 percentage <15% or CD4 count < 350 cps/mm^3^ above 35 months old.

^b^CD4 count based on *n* = 218; CD4 percentage based on *n* = 201.

^c^Viral load based on *n* = 184.

^d^Main reason for initiation as indicated by clinician.

**Table 3:** Nutritional status of the “Monitoring of Antiretroviral Therapy in Children” cohort participants at presentation.

		*n* = 306
Underweight (WAZ <−2 SD)	<5 years old	42.7 (34.6–50.7)
	5–12 years old	24.5 (15.2–33.7)
Severe underweight(WAZ <−3 SD)	<5 years old	26.1 (18.9–33.3)
	5–12 years old	12.8 (5.5–20.0)

Stunting (HAZ <−2 SD)	<5 years old	62.0 (53.9–70.1)
	5–12 years old	39.0 (30.4–47.5)
Severe stunting (HAZ <−3 SD)	<5 years old	40.0 (31.8–48.2)
	5–12 years old	18.4 (11.5–25.3)

Wasting (WHZ <−2 SD)	<5 years old	21.7 (14.9–28.4)
	5–12 years old	NA
Severe wasting (WHZ <−3 SD)	<5 years old	7.0 (2.7–11.3)
	5–12 years old	NA

BMI-for-age *z*-score (<−2 SD)	<5 years old	16.3 (10.2–22.3)
	5–12 years old	8.6 (3.6–13.7)

Midupper arm circumference *n* (%)^a^	≤13.5 cm	55 (37.9)

Data are presented as percentage with 95% confidence interval (CI) unless otherwise indicated. No significant differences were found between clinical sites. Reference data used are WHO Anthro version 3.2.2, January 2011 for age 0–5 and Reference 2007 for age 5–19 [13, 14, 21]. 33 *z*-scores were excluded from analysis because of biological implausibility (WAZ *n* = 9, HAZ *n* = 19, WHZ *n* = 3, and BMI-for-age *n* = 2).

^a^Midupper arm circumference only applicable for children 1–5 years old (*n* = 148). WAZ: weight-for-age *z*-score, HAZ: height-for-age *z*-score, WHZ: weight-for-height z-score, and BMI: body mass index (in kg/m^2^).

**Table 4:** Risk factors for late disease stage at presentation (WHO stage 3 or 4).

		Univariate analysis			Multivariate analysis		
		OR	95% CI	*P* value	OR	95% CI	*P* value
Sex	Female	1					
	Male	0.99	0.56–1.74	0.963			

Age group	5–12 years old	1			1		
	2–5 years old	1.20	0.60–2.41	0.612	1.90	0.85–4.22	0.117
	0–2 years old	1.45	0.72–2.92	0.303	2.83	1.23–6.50	0.014

Current living situation	With both parents	1			1		
	With one parent	1.39	0.71–2.73	0.340	2.05	0.96–4.35	0.062
	With siblings/relatives/other caregiver/in institution	2.46	1.14–5.31	0.021	3.93	1.65–9.38	0.002

Primary caregiver	Mother	1					
	Father	0.80	0.22–2.94	0.738			
	Grandparent	3.07	1.06–8.92	0.039			
	Other relative/friend	0.92	0.45–1.91	0.826			
	Institution	3.09	0.54–17.64	0.203			

Mother's health status	Healthy	1					
	Sick	1.66	0.49–5.60	0.416			
	Deceased	1.59	0.71–3.58	0.260			
	Unknown	1.10	0.28–4.24	0.894			

Father's health status	Healthy	1					
	Sick	1.27	0.43–3.74	0.660			
	Deceased	1.78	0.82–3.85	0.142			
	Unknown	2.17	0.81–5.81	0.122			

Highest level of education of the caregiver	Illiterate	1					
	Literate/primary school	0.77	0.32–1.83	0.550			
	Secondary school	0.55	0.21–1.44	0.223			
	Postsecondary school	0.49	0.17–1.45	0.197			

Occupation of the caregiver	Wage-employed	1			1		
	Self-employed	1.82	0.78–4.26	0.166	2.50	1.00–6.28	0.051
	None/at home/student	3.24	1.43–7.34	0.005	4.26	1.75–10.35	0.001

PMTCT experienced	Yes	1			1		
	No	5.07	1.24–20.79	0.024	5.66	1.21–26.51	0.028

Time between HIV+ diagnosis and ART initiation	0–31 days	1					
	>30 days	0.93	0.50–1.73	0.818			

Transportation time	<1 hour	1					
	1 hour or more	0.85	0.45–1.58	0.606			

Transportation costs	1st quartile (UGX^a^ 500–1500)	1			1		
	2nd quartile (UGX 1500–3000)	1.07	0.51–2.23	0.857	0.96	0.43–2.14	0.915
	3rd quartile (UGX 3000–4000)	0.89	0.34–2.33	0.818	0.85	0.30–2.38	0.756
	4th quartile (UGX 4000–15000)	2.09	0.85–5.15	0.108	2.51	0.92–6.85	0.072

Waiting time at clinic	<2 hours	1					
	2 hours or more	0.83	0.35–1.98	0.676			

Multilevel univariate and multivariate logistic regression analysis with random intercepts to examine factors associated with late disease at presentation (WHO stage 3 or 4), accounting for clustering of observations within sites.

^a^UGX 500 *≈* $0.18; UGX: Uganda Shilling.
